# Prevalence and Characteristics of Sleep Disorders in Children Aged 7–17: Insights from Parental Observations at the Dental Office

**DOI:** 10.3390/children11050609

**Published:** 2024-05-20

**Authors:** Montserrat Diéguez-Pérez, Laura Burgueño-Torres, Guillermo Reichard-Monefeldt, Fanny Esther Tapia-Sierra, Jesús Miguel Ticona-Flores

**Affiliations:** 1 Department of Preclinical Dentistry, Faculty of Biomedical and Health Sciences, Universidad Europea de Madrid, Villaviciosa de Odón, 28670 Madrid, Spain; 21212424@live.uem.es (G.R.-M.); jesus.ticona.f@upch.pe (J.M.T.-F.); 2 Dental Clinical Specialties Department, Faculty of Dentistry, Complutense University of Madrid, 28040 Madrid, Spain; lbtorres@ucm.es (L.B.-T.); ftapia01@ucm.es (F.E.T.-S.)

**Keywords:** sleep disorders, children, etiology, sleep questionnaires, screening, sleep questionnaires

## Abstract

Sleep disorders (SD) in children is a topic of great relevance due to their impact on a child’s general health. This has led us to study their prevalence and the factors that disturb them in the developing population. Using a validated Likert-type questionnaire, different behaviors observed by the parents during the different phases of their children’s sleep were evaluated during the last 6 months. A total of 206 children between the ages of 7 and 17 who attended a dental office participated in the study. The prevalence of SD was 47.6%. There were no significant differences regarding the SD in relation to sex (*p* = 0.796). The mean total score for children aged 7 to 11 years old was 42.3 (±14.25) compared to 45.44 (±15.51) for the group consisting of children aged 12 to 17 years old, reporting a statistically significant difference among both age groups (*p* = 0.01). The most frequent disorder was related to initiating and maintaining sleep (64.9%) while the least prevalent were the respiratory sleep disorders (27.2%). Given the high prevalence of these disorders, it is necessary to intercept them during childhood and establish educational guidelines in this regard throughout primary care.

## 1. Introduction

Sleep is defined as a reversible, cyclical, active, essential and complex state of unconsciousness that plays a primary role in several spheres of child development such as neurocognitive as it facilitates learning and consolidates memory [1], while also playing a primary role in the emotional and behavioral spheres [2,3,4]. It is a relevant process in the anatomical and physiological architecture of the central nervous system and is already present in the fetus, even though its structure and physiology are modified according to the maturation of this nervous system [5]. Sleep habits are influenced by biological, ethnic, ecological, social and cultural factors [6,7].

Sleep disorders are a common problem during childhood, including sleepwalking, insomnia, hypersomnia, movement disorders, somniloquy, confused awakenings, night terrors, nocturnal enuresis, bruxism, circadian and respiratory disorders [8,9]. Each of them can affect the child’s general health status, can be associated with medical comorbidities [6,10,11] and even be a risk factor for mental disorders [12,13]. Sleep disorders can be classified into six groups: insomnia, hypersomnia, parasomnia, movement disorders, circadian disorders and respiratory disorders [4,5,11]. In some cases, a bidirectional relationship can be observed between these sleep disorders and other childhood medical and mental health problems [14], favoring the development of pathologies such as anxiety, depression and autism spectrum disorders during adolescence [15,16]. Pediatricians and pediatric dentists must routinely evaluate the study of sleep–wake function as an improvement in children’s quality of life, hence the relevance of its evaluation to address them early in life. Only in this way could disorders such as daytime sleepiness, behavioral problems and learning disorders associated with poor academic performance be averted early during childhood. Addressing sleep disorders early on in life might also help avoid some traffic accidents during adolescence [11,17].

In children, sleep duration and habits vary widely between countries with different cultural origins. The recommended number of hours of sleep varies depending on age, preschool children (3–5 years) need between 10 and 13 h of sleep, schoolchildren (6–13 years) between 9 and 11 h and adolescents (14–17 years) require between 8 and 10 h of sleep [6]. Currently, parents report between 10 and 70% of sleep disturbances in pediatric age groups [4,5,15]. In Spain, sleep disorders in children are barely studied [18,19,20], as only three investigations refer to them and one of the most recent was carried out in the Autonomous Community of Catalonia; however, there are no recent studies in other Spanish populations [20]. Based on the data obtained in other investigations, we establish as a working hypothesis that less than half of the study population will present some sleep disorder. At the same time, two age groups were established taking into account the educational level of the developing patient.

Based on all these premises, our objective is to study the prevalence of sleep disorders related to initiating and maintaining sleep (DIMS), sleep breathing disorders (SBD), arousal disorders (AD), sleep–wake transition disorders (SWTD), excessive somnolence disorders (SD) and sleep hyperhidrosis (SHY).

## 2. Materials and Methods

To achieve the proposed objectives, a descriptive, observational and cross-sectional study was designed.

### 2.1. Ethical Aspects

This research received approval from the Ethics and Research Committee of the European University of Madrid with internal code 2024-427. During the extent of the study, the ethical principles of the Declaration of Helsinki were considered, as well as the Spanish legal provisions, specifically law 15/1999 on data protection, by which privacy and confidentiality of the data obtained during the research process were guaranteed at all times. Likewise, this study complies with the provisions of Law 41/2002 regulating patient autonomy and rights and obligations regarding information and clinical documentation.

### 2.2. Population and Eligibility Criteria

The universe of the study consisted of healthy developing children of both sexes who requested a dental check-up appointment in various private dental centers in the Community of Madrid and Castilla-La Mancha. Healthy children between the ages of 7 and 17 were included as long as their parents agreed to voluntarily sign an informed consent followed by the assent of the participating children. Two age groups were established, one of them for 7–11-year-olds and a second group consisting of 11–17-year-old children, based on the level of education. Patients whose parents were not able to provide the requested data in relation to sleep disorders, in addition to patients who take any medication that may excessively or deficiently affect the normal sleep period were excluded.

### 2.3. Sample Size

To calculate the sample size, the formula for estimating proportions without a sampling frame extracted from Serdar et al. was applied [21]. A significance level of 0.05, a power of 80% and a precision of 95% were established. The prevalence of sleep disorders was taken as 4.24% for the Spanish population, according to the results reported by Pagerols et al. [20]. The minimum participants required consisted of 63 children.

### 2.4. Variables and Procedures for Their Collection

In this research, sociodemographic study variables, data referring to the age (in years and months) and sex of the pediatric patient were used. The variables related to sleep disorders present during the last 6 months included initiation of sleep and maintenance disorders, respiratory sleep disorders, sleep arousal disorders, sleep–wake transition disorders, excessive somnolence disorders and excessive hyperhidrosis.

**Figure 1:**
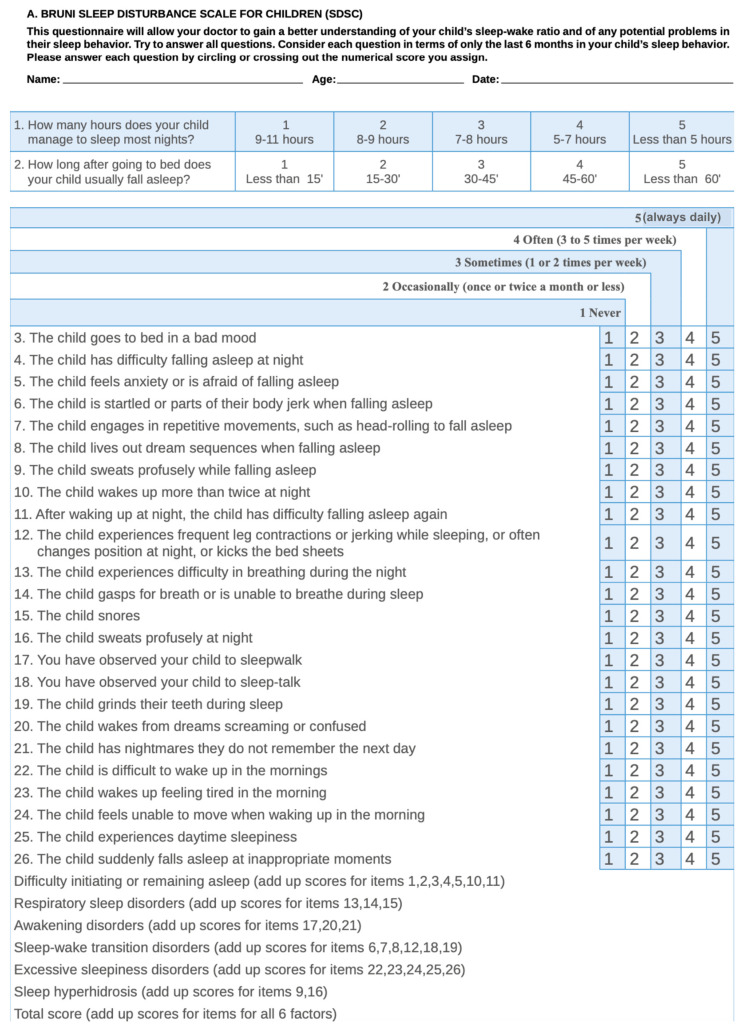
Questionnaire used to determine the child’s sleep disturbances [22].

Upon arrival at the dental clinic, the parents and the child were informed of the possibility of cooperating in the research; subsequently, the signing of the informed consent and collection of the patient’s sociodemographic data took place. The data in relation to possible sleep disorders that the children could present were compiled by filling in the validated sleep disturbance scale for pediatric population (SDSC) proposed by Bruni et al. [22] and validated [20] (Figure 1), widely used in the scientific literature and tested in the Spanish language. This scale is a questionnaire consisting of 26 items related to the possible factors that could interfere with the child’s restful sleep. The main researcher, an experienced pediatric dentist, was the one who briefly explained the content of the questionnaire, answering the doubts raised by the parents during the response process. The scale uses five response options, such that 1 indicates “never happens,” 2 “occurs 1 or 2 times a month,” 3 “occurs 1 or 2 times a week,” 4 “occurs 3 or 5 times a week.”, 5 indicates “occurs daily.” Higher response values reflect greater severity of the clinical manifestations of these disorders. The first two questions measure total sleep time and latency concerning sleep onset. Questions 1, 2, 3, 4, 5, 10 and 11 are related to disorders initiating and maintaining sleep (DIMS), items 13, 14 and 15 correspond to sleep breathing disorders (SBD), questions 17, 20 and 21 correspond to arousal disorders (AD), items 6, 7, 8, 12, 18 and 21 correspond to sleep–wake transition disorders (SWTD).), while questions 22, 23, 24, 25 and 26 correspond to excessive somnolence disorders (SD) and items 9 and 16 correspond to sleep hyperhidrosis (SHY).

### 2.5. Statistic Analysis

The statistical analysis of the data was carried out using IBM SPSS Statistics software platform, version 25. The qualitative variables that correspond to the sociodemographic characteristics were described using relative and absolute frequencies. On the other hand, the quantitative variables, which represent the scores of each item, were described through measures of central tendency such as the mean, median and standard deviation. The scores for each item were evaluated based on sex and age range using the Mann–Whitney U test because the normality criteria of the quantitative variables were not met (Kolmogorov–Smirnov, *p* > 0.05). The scores were categorized as presence or absence of sleep disorder, following the recommendations of Bruni et al. [22], and were described by relative and absolute frequencies. These frequencies were compared according to sex and age range using the Chi-square test. A significance level of *p* < 0.05, a power of 90% and a precision of 95% were established.

## 3. Results

### 3.1. Sample Description

In this study, 206 questionnaires were analyzed corresponding to 96 (46.6%) males and 102 females (49.5%), while eight patients preferred not to report their gender. The average sample age was 11.18 years (±2.91). The mean ages divided by age groups are detailed in Table 1. When evaluating the composition of the sample, no significant differences were found between the number of girls and boys who participated [X2(1) = 0.182; *p* = 0.182], nor were differences reported between the two age groups [X2(1) = 1.573; *p* = 0.210].

### 3.2. Scores Gathered from the Sleep Disturbance Scale Survey

**Figure 2:**
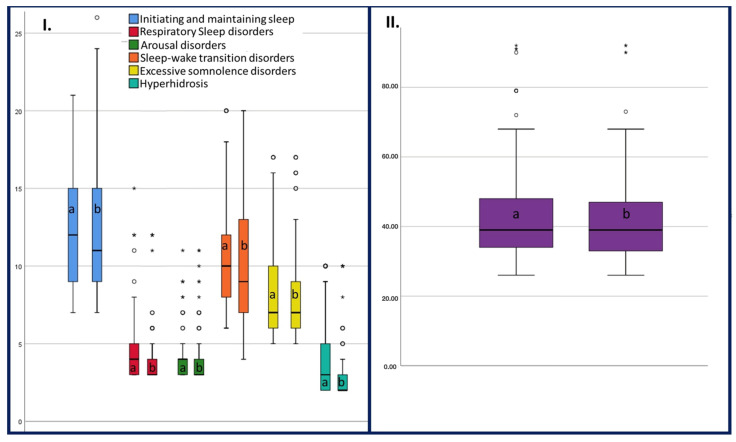
Box plot. (**I**) Score representation for each item by sex. (**II**) Total Bruni score representation. a—male; b—female.

The questionnaire yielded a mean total score of 43.69 points (±14.89) and a median score of 39.00 points. When gender was compared, the mean score did not differ significantly (z = −0.930, *p* = 0.352) between boys (44.93 ± 16.10) and girls (42.54 ± 14.13). The differences are illustrated in Figure 2 and the corresponding mean scores for each item are reported in Table 2.

### 3.3. Frequency of Sleep Disturbances

The frequency of participants with a sleep disturbance (score greater than 40) was 47.6% (98). The overall frequency of sleep disturbances did not report statistically significant differences (X^2^ = 0.67, *p* = 0.796) between boys (23.2%) and girls (23.7%). The frequency of alterations per item is reported in Table 3. 

### 3.4. Comparison between Both Groups

Group A corresponding to 7 to 11-year-old children presented an average total score of 42.3 points (±14.25), compared to 45.44 points (±15.51) for the 12 to 17-year-old children from group B. These results presented a statistically significant difference (z = −2.593; *p* = 0.01). The comparisons of each item are expressed in Table 4. The statistically significant differences indicate that age group B (12 to 17 years) presents higher mean scores in initiating and maintaining sleep and excessive somnolence disorders.

The group of 7–11 years-old children presented a frequency of sleep disturbance of 21.4% (*n* = 44); however, the group of 12–17-year-old children presented a frequency of 26.2% (*n* = 54), these results were statistically significant (X^2^ = 6.76, *p* = 0.009); OR = 2.086 [95% CI: 1.195–3.644]. The comparisons of each item are expressed in Table 5. It should be noted that group B showed twice the risk (OR = 2.5 [95% CI: 1.36–4.8] and OR = 2.03 [95% CI: 1.16–3.57]) when compared to group A (younger children) for the items of initiation and maintenance of sleep and excessive somnolence disorders.

## 4. Discussion

Most sleep disorders during childhood could be routinely detected through simple inquiries directed to the parents during pediatric and pediatric dental check-ups. Age is a factor to take into consideration since, according to Babcock [23], at the age of 6–12 years, sleep habits are usually already established and if these are not adequate, they could worsen the sleep quality. Circadian rhythm disorders, restless legs syndrome or obstructive sleep apnea (OSA) can exacerbate insomnia. Specifically, OSA is considered the most serious form of all sleep breathing problems, which are common in the pediatric population. Some researchers point to age and body mass index (BMI) as possible risk factors [24]. Common complaints related to sleep disturbances include difficulty initiating or maintaining sleep, abnormal behavior or movements, snoring or abnormal breathing and excessive daytime sleepiness [4].

A large part of the research on sleep disorders is based on populations with established medical comorbidities, perhaps because it is expected that in these circumstances, the prevalence of the disorders will be higher and preventive or improvement measures can be taken. In the healthy pediatric population, preschool children were the most commonly studied and this limits the comparability of our results.

### 4.1. Validated Questionnaires

The Pediatric Sleep Questionnaire (PSQ), Sleep Self-Report Scale (SSRS), Pediatric Sleep Survey Instrument (PSSI), Children’s Sleep Habits Questionnaire (CSHQ) and Sleep Disturbance Scale for Children (SDSC) are the different measurement tools used to determine the frequency of sleep disorders in different investigations. This plurality interferes with the comparability of the results among the studies. According to some research, the prevalence of sleep disorders differs depending on the questionnaire used. In fact, they are more prevalent when the CSHQ scale is used compared to the SDSC scale [25]. In this regard, and from our point of view, it is a confusing factor to take into account.

The SDCS is an accurate scale even though it is translated into different languages. There are English, French, Spanish, Italian, Turkish and Persian versions. In our case, the Spanish version was already validated. It was proposed by Bruni et al. as a useful tool to evaluate sleep disturbances in school-aged children and has been widely employed since 1996. It was designed as a standardized measure of sleep disorders, is straightforward for doctors and researchers, and its purpose was to be able to generate a database in different populations and thus be able to define normality and identify different typologies and specific areas of sleep disorders. According to these researchers, childhood sleep disorders are not independent entities but are related to each other [22].

### 4.2. Prevalence of Sleep Disturbances

The most striking aspect of this research is the high prevalence of sleeping disorders in the study population, who did not present any prior medical problems. We believe that this may perhaps be due to the greater width of the age range studied and the fact that all children of our sample requested dental care since the majority of previous research was carried out studying the general pediatric population. In this regard, the frequency of sleep disorders in a population of 1903 5 to 15-year-old Turkish children was 4.15%, with the most common sleep disorder being sleep hyperhidrosis [26]. In a study conducted with 262 Dutch children with ages ranging from 7 to 12 and a mean age of 8.5 years, 25% of the sample presented at least one sleep-related problem [27]. According to the study by Gao et al. [28], the prevalence of sleep disorders among 1336 Chinese children of younger ages (3–6 years) was 14.29%. The highest frequency of these disorders were alterations related to the sleep–wake transition since 42.81% of the sample presented movements of the extremities, 18.11% had bruxism and 16.39% spoke during sleep. Only 19.61% of the children snored and 3.74% developed apnea, both of these percentages are related to respiratory disorders. Hyperhidrosis was present in 12.57% of the children. Night awakenings or sleep initiation and maintenance disorders occurred in 11.60% of the sample. Furthermore, nightmares and sleepwalking or arousal disorders were presented in 8.46% and 3.29% of the cases, respectively. In addition, 6.89% of the children presented enuresis, an aspect that is not reflected in the SDSC but would be another factor to take into account to evaluate the prevalence of sleep disorders. The prevalence of body movements, snoring, sweating, nocturnal awakenings, nightmares, enuresis, sleep apnea and sleepwalking between different ethnic groups were significantly different (*p* < 0.05). These studies reveal the great variability involving the prevalence of sleep disorders. Aspects to take into account that determine the prevalence of sleep disorders are the characteristics surrounding the birth of the child, since according to Brockmann et al. [29], sleep disorders can originate during early stages of life. Children born prematurely show a greater risk of developing long-term sleep disorders. This aspect invites us to consider the establishment of preventive measures to optimize sleep habits from early childhood.

### 4.3. Research on the Spanish Population

Comparatively with the studies carried out in our country, Quinteros-Hinojosa et al. [18] studied the prevalence of sleep disorders in a sample of 6 to 12-year-old children, most being 9 years old on average. The frequency of these disorders in girls was much higher (69.8%) compared to that of boys (30.2%); however, in our study, no statistically significant differences were found in any of the disorders. The prevalence of sleep disorders in the total sample was 34.9% and is close to our prevalence, possibly because the study population in both studies requested dental care. The most prevalent disorders according to Hinojosa et al. were sleep–wake transition disorders with a measurement of 53.5% vs. 61.7% in our research, followed by sleep initiation and maintenance disorder with 44.2% compared to 69.4% in our study. Respiratory sleep disorders occurred less frequently than in our study (39.5% vs. 27.2%). However, excessive somnolence disorders (34.9% vs. 41.7%) and sleep hyperhidrosis (32.6% vs. 44.7%) were higher in our study. Another more recent Spanish investigation conducted by Pagerols et al. [20] was carried out with 6–16-year-old children (N = 2733) and 4.24% of the sample presented at least one sleep disorder. In addition, 5.82% of the cases presented disorders of excessive somnolence, 5.27% exhibited sleep–wake transition disorders and 5.09% displayed disorders when initiating and maintaining sleep, which was the most frequent disorder in our study (69.4%). The lower percentage is striking compared to the results of our study and we believe it is due to the samples being from different geographic regions. According to these authors, the socioeconomic and cultural factors affected the results of their study. In addition, Diéguez-Pérez et al. [19] studied sleep disorders in a Spanish preschool population but focused on the bruxism habit, which is an alteration related to the sleep–wake transition disorder. The prevalence of sleep disorders in the 343 children who participated in the study was 28.9%, a lower percentage than that obtained in our study. Perhaps age was an influential factor in this difference. Tachibana et al. related this parafunction to obstructive sleep apnea, reporting a 21% prevalence of sleep bruxism among a sample of 6023 Japanese children between the ages of 2 and 12 [30].

### 4.4. Respiratory Sleep Disorders

Most research focuses solely on respiratory sleep disorders ranging from snoring to obstructive sleep apnea. Aroucha Lyra et al. determined a prevalence of 33.3% in 390 7 to 8-year-old Brazilian children, which was significantly associated with occlusal alterations such as anterior open bite (*p* = 0.002; OR 95% CI: 2.34) and posterior crossbite (*p* = 0.014; OR 95% CI: 2.79). According to Alencar et al. [31], nightmares and snoring are associated with nocturnal bruxism in Brazilian children with an average age of 5.33 (±1.10) years. In fact, the study found that those children who suffered nightmares were 18 times more likely to suffer from bruxism. On the other hand, drooling, sleepwalking, waking up during the night and nocturnal enuresis did not demonstrate an association with bruxism. Bruxist children tend to wake up feeling tired, with difficulty getting out of bed and with a low mood. Furthermore, in a study conducted with a total of 1350 Saudi children with a mean age of 9.2 ± 1.8 years, 14.4% had a snoring prevalence. According to these authors, children with oral breathing, adenoiditis, otitis media and orofacial symptoms such as onychophagia, bruxism and pain in the temporomandibular joint when waking up had a greater risk of suffering from respiratory disorders during sleep (*p* < 0.0001) than children without these conditions [32]. In this regard, the prevalence of snoring was significantly higher in asthmatic children (35.5%) than in the control group (15.7%) [33]. Another investigation that was carried out with 65 Saudi children with a mean age of 9.75 (±2.60) years determined a 12.3% prevalence rate of breathing disorders during sleep [34]. A multicenter study carried out simultaneously between Italy and Spain determined a 9.7% prevalence of respiratory sleep disorders [35]. Allergic rhinitis, asthma, tonsillar size, class I molar relationship, posterior crossbite and overweight and obesity are also risk factors to take into account [34,36,37]. Sánchez et al. [38] analyzed 564 questionnaires corresponding to a sample of 5 to 9-year-old Chilean children with an average age of 6 years. The prevalence of sleep breathing disorders differed depending on the geographical region to which they belonged, being 17.7%, 6%, 28.7% and 36.4% (*p* = 0.001). Symptoms of rhinitis during the last 12 months (OR 4.79; 95% CI 2.20–10.43) and lower maternal educational level (OR 3.51; 95% CI 1.53–8.02) were considered predictors of these disorders. According to Sakamoto et al. and after assessing the questionnaires of 17,769 Japanese children, respiratory sleep disorders decreased with age [39]. Perhaps, for this reason, the prevalence of these disorders was much higher compared to all these investigations. All these aspects should make us reflect when establishing preventive and therapeutic measures for sleep disorders in the pediatric population.

### 4.5. Other Considerations: Limitations and Strengths

Sleep disorders during childhood are an increasingly common public health problem [8]. For this reason, we insist on the importance of promoting good sleep habits from an early stage and to do so, it is essential to educate parents and their children about their importance in children’s health. Only in this way could we reinforce the avoidance of bad habits that in the future could constitute risk factors regarding the child’s quality of life. In addition to these practices, a sleep diary, home videos and clock-shaped devices with a speedometer that reflect the movement of the extremities during sleep could be used as preliminary tools. The results gathered from these devices could be a first step for their evaluation and assessment of the need for referral to a sleep specialist for a polysomnographic analysis and other complementary tests if a specific sleep disorder, such as obstructive sleep apnea and narcolepsy, is suspected.

With this study, we want to highlight the role of the pediatric dentist in the early diagnosis of these sleep disorders through the questionnaires and home tools mentioned above.

Although the process of the data obtained does not derive from the observation of a medical professional, which represents a limitation of the study, we believe that it can also be considered as an alarm signal to refer these patients toward a definitive diagnostic path and multidisciplinary treatment. Another aspect to take into account and that we also consider a limitation is the lack of knowledge of the socioeconomic characteristics of the study population and their daily habits since this would allow them to be alerted about the relevance of modifying them in order to try to reduce the frequency of these disorders. One of the strengths is the measurement tool used, as it is a resource that allows the presence of these alterations to be quickly and easily detected.

## 5. Conclusions

The prevalence of sleep disorders in the study population was 47.6%, with no significant differences in relation to sex. The most common sleep disorder was initiating and maintaining sleep with a 69.4% frequency, followed by sleep–wake transition disorders (61.7%), sleep arousal disorders (48.5%), sleep hyperhidrosis (44.7%), excessive somnolence disorders (41.7%) and respiratory sleep disorders (27.2%). The results of this study reveal to us the extent to which the quality of life of the developing population may be compromised. In this regard, we should reflect on what risk factors favor the presence of these disorders.

Knowing the prevalence and identifying sleep disorders allows for early intervention and prevention of long-term adverse effects. Outpatient sleep analysis should be a routine test. Educating parents and their children about the relevance of sleep is essential, as is routine outpatient sleep analysis. Treatment must be multidisciplinary since there are multiple possible etiological factors.

## Figures and Tables

**Table 1:** Average age of each group by gender.

	Age		Gender			
Groups	Total Participants		Males		Females	
	Mean	SD	Mean	SD	Mean	SD
A	9.11	1.66	8.98	1.67	9.16	1.7
B	13.66	2.01	14.03	1.8	13.42	2.14

SD: standard deviation; A: children aged 7 to 11 years; B: children aged 12 to 17 years.

**Table 2:** Mean scores divided by sex for each item on the Sleep Disturbances Scale.

	Mean	SD	Median	Sex	Mean	SD	Median	*p*-Value
Initiating and maintaining sleep	12.49	4.39	11.50	M	12.38	4.16	12.00	0.990
				F	12.59	4.74	11.00	
Respiratory Sleep disorders	4.25	2.16	3.00	M	4.41	2.29	4.00	0.127
				F	4.05	2.02	3.00	
Arousal disorders	4.06	1.66	3.00	M	4.11	1.61	4.00	0.359
				F	4.05	1.74	3.00	
Sleep–wake transition disorders	10.85	4.73	10.00	M	11.15	5.21	10.00	0.703
				F	10.61	4.40	9.00	
Excessive somnolence disorders	8.51	3.84	7.00	M	8.81	4.59	7.00	0.820
				F	8.24	3.09	7.00	
Hyperhidrosis	3.53	2.43	2.00	M	4.07	2.78	3.00	0.003 *
				F	3.01	1.96	2.00	

SD: standard deviation; M: males; F: females; *p*: Mann–Whitney U test *p*-value significance level; *: statistically significant comparison (z = −2.93).

**Table 3:** Frequency of each sleep disorder depending on gender.

Presence of Sleep Alteration *	n	% ^1^	Sex	n	% ^2^	*p*-Value
Initiating and maintaining sleep (score > 10)	143	69.4	M	66	33.3	0.985
			F	70	35.4	
Respiratory Sleep disorders (score > 4)	56	27.2	M	31	15.7	0.061
			F	21	10.6	
Arousal disorders (score > 4)	100	48.5	M	49	24.7	0.575
			F	48	24.2	
Sleep–wake transition disorders (score > 9)	127	61.7	M	58	29.3	0.846
			F	63	31.8	
Excessive somnolence disorders (score > 8)	86	41.7	M	39	19.7	0.827
			F	43	21.7	
Hyperhidrosis (score > 3)	92	44.7	M	49	24.7	0.070
			F	39	19.7	

Presence of alteration *: where the score that is considered an alteration of each item is specified; n: Sample; % ^1^: Percentage taking into account all 206 participants; F: females; M: males; % ^2^: Percentage taking into account 198 participants who reported their sex; X^2^: significance level.

**Table 4:** Comparison of the Sleep Disturbances Scale scores by groups of different age ranges.

	Group	Mean	SD	Median	Maximum Value	Minimum Value	*p*-Value
Initiating and maintaining sleep	A	11.86	4.33	11.00	29.00	7.00	0.003 *
	B	13.24	4.38	12.50	29.00	7.00	
Respiratory Sleep disorders	A	4.26	2.23	3.00	15.00	3.00	0.889
	B	4.24	2.08	3.50	12.00	3.00	
Arousal disorders	A	4.10	1.64	4.00	11.00	3.00	0.702
	B	4.02	1.68	3.00	11.00	3.00	
Sleep–wake transition disorders	A	10.59	4.53	10.00	26.00	4.00	0.785
	B	11.16	4.97	9.50	26.00	6.00	
Excessive somnolence disorders	A	7.99	3.20	7.00	23.00	5.00	0.013 **
	B	9.13	4.42	7.00	23.00	5.00	
Hyperhidrosis	A	3.44	2.27	2.00	10.00	2.00	0.403
	B	3.64	2.63	2.00	10.00	2.00	

SD: standard deviation; A: children aged 7 to 11 years; B: children aged 12 to 17 years; *: Mann–Whitney U significance (z = −2.95); **: Mann–Whitney U significance (z = −2.48).

**Table 5:** Frequency of sleep disturbances compared by groups of different age ranges.

	Group	n	%	*p*-Value	OR	95% CI	
Initiating and maintaining sleep	A	68	33.0	0.003 *	2.554	1.360	4.797
	B	75	36.4				
Respiratory Sleep disorders	A	30	14.6	0.888	1.045	0.565	1.935
	B	26	12.6				
Arousal disorders	A	53	25.7	0.702	1.113	0.643	1.927
	B	47	22.8				
Sleep–wake transition disorders	A	70	34.0	0.784	0.924	0.526	1.624
	B	57	27.7				
Excessive somnolence disorders	A	38	18.4	0.013 **	2.032	1.158	3.567
	B	48	23.3				
Hyperhidrosis	A	53	25.7	0.402	0.789	0.454	1.372
	B	39	18.9				

SD: standard deviation; A: children aged 7 to 11 years; B: children aged 12 to 17 years; *p*-value: X^2^ significance level; *: X^2^ significance = 8.757; **: X^2^ significance = 6.17; OR: odds ratio; CI: confidence interval.

## Data Availability

Data are contained within the article.
